# A Meta-Analysis on the Impact of the COVID-19 Pandemic on Cutaneous Melanoma Diagnosis in Europe

**DOI:** 10.3390/cancers14246085

**Published:** 2022-12-10

**Authors:** Konstantinos Seretis, Nikolaos Bounas, Georgios Gaitanis, Ioannis Bassukas

**Affiliations:** 1 Department of Plastic Surgery, School of Health Sciences, University of Ioannina, 45100 Ioannina, Greece; 2 Department of Skin and Venereal Diseases, Faculty of Medicine, School of Health Sciences, University of Ioannina, 45100 Ioannina, Greece

**Keywords:** COVID-19, melanoma, skin cancer, diagnosis, Europe, meta-analysis

## Abstract

**Simple Summary:**

Malignant melanoma is the most aggressive type of skin tumor, with prompt diagnosis constituting the cornerstone of an optimal management plan. The coronavirus pandemic, however, has altered the global healthcare landscape, disabling screening services and tumor surveillance processes. The aim of this meta-analysis was to measure the repercussions of the adjustments implemented for the containment of the COVID-19 pandemic and to quantify the resulting tumor burdens in melanoma patients in the European continent. We managed to pinpoint that clinically more advanced, thicker melanomas with higher ulceration rates occurred in the post-COVID era. The lockdown period impacted mostly the diagnosis of melanomas. These outcomes stress the importance of enhanced and optimized melanoma screening programs and pave the way for future research to address the impact of the pandemic on melanoma treatment efficacy in terms of survival rates.

**Abstract:**

The COVID-19 pandemic has been the epicenter of healthcare attention globally for the past two years, and large-scale adaptations in healthcare provision have been required. This study aimed to investigate the impact of the pandemic and the resulting lockdowns on cutaneous melanoma diagnosis and tumor burdens in Europe. A relevant literature search in electronic databases was conducted from inception to September 2022. The inclusion criteria were: controlled studies published in a peer-reviewed journal evaluating cutaneous melanoma in Europe and reporting data on melanoma characteristics from diagnoses. The quality of studies was evaluated using the Cochrane ROBINS-I tool for assessing bias in non-randomized studies. Meta-analysis was conducted utilizing a random effects model to synthesize the data. A total of 25 studies involving 32,231 patients were included in the data analysis models. Statistically significant increases in mean Breslow thickness (0.29 mm (0.03–0.55 mm)), ulceration rates (OR = 1.66 (1.29–2.13)), and resultant tumor staging were observed in the PostCovid group, with subgroup analysis revealing that lockdown-derived data were responsible for this trend. This meta-analysis reported on the impact of COVID-19 restrictions on melanoma diagnosis in Europe, emphasizing the higher tumor burden and disease progression state provoked by healthcare adaptations in the pandemic period.

## 1. Introduction

The COVID-19 pandemic has been the epicenter of healthcare attention globally for the past 2 years. Shortly after the formal declaration of the pandemic by the World Health Organization in March 2020, most countries worldwide imposed harsh restrictions in an effort to impede the accelerating infection rates. The situation in Europe was no different, since most countries enforced complete lockdowns in almost identical time periods throughout 2020–2021. The direct outcome was an unprecedented crisis which dealt a major socioeconomic blow and had detrimental effects on the general population’s psychological health and well-being [1,2].

Healthcare services had to redirect resources in order to address the immense workload imposed by the surging viral infections, while access to medical facilities was restricted as part of quarantine measures. Specifically, elective surgical procedures were suspended to conserve hospital and intensive care unit (ICU) beds, as well as to protect patients and medical professionals from in-hospital transmission of the virus [3]. Significant delays were witnessed for time-sensitive oncologic operations, which undoubtedly was detrimental to the survival of cancer patients. This has been shown in a recent meta-analysis that confirmed the association between delay of surgery and increased mortality [4].

Malignant melanoma (MM) is the most aggressive skin malignancy and requires prompt diagnosis and curative oncologic resection to guarantee optimal survival of patients [5]. It is the most rapidly increasing cancer in the white population worldwide, with an estimated annual increase rate between 3% and 7% [6]. Despite this fact, the strategy of deferral for low-priority tumors in areas manifesting a high prevalence of infections has been supported by relevant scientific organizations, such as the National Comprehensive Cancer Network (NCCN) and the British Association of Plastic Surgery [7,8]. This decision was made as part of the effort to ensure the availability of medical resources for the control of the pandemic. Similarly, dermatologic outpatient examinations and screening programs were severely disrupted as appointments were systematically canceled by both patients and providers [5].

Multiple reports worldwide have addressed the decreased number of melanoma diagnoses during the pandemic. The aim of this meta-analysis was to investigate the impact of the pandemic and the resulting lockdowns on cutaneous melanoma diagnosis in Europe and provide evidence pertaining to the impact of the employed health strategies on the melanoma burden, as assessed by the recognition and treatment of more advanced tumors.

## 2. Materials and Methods

A meta-analysis was conducted using a predetermined protocol established according to the *Cochrane Handbook*’s recommendations [9]. The review adhered to the updated PRISMA (Preferred Reporting Items for Systematic Reviews and Meta-Analyses) guidelines (Table S1) [10]. The review protocol was registered with PROSPERO (registration no. CRD42022364051)

### 2.1. Search Strategy

An electronic literature search in MEDLINE (PubMed), Scopus, the Cochrane Library and US National Institutes of Health Ongoing Trials Register electronic databases was conducted from inception to September 2022. The string search (“cutaneous melanoma”) and (“COVID”) was applied. No time and language restrictions were applied. This search was supplemented by a review of reference lists of potentially eligible studies and a manual search of key journals in the fields of dermatology and plastic surgery.

### 2.2. Eligibility of Relevant Studies

**Figure 1:**
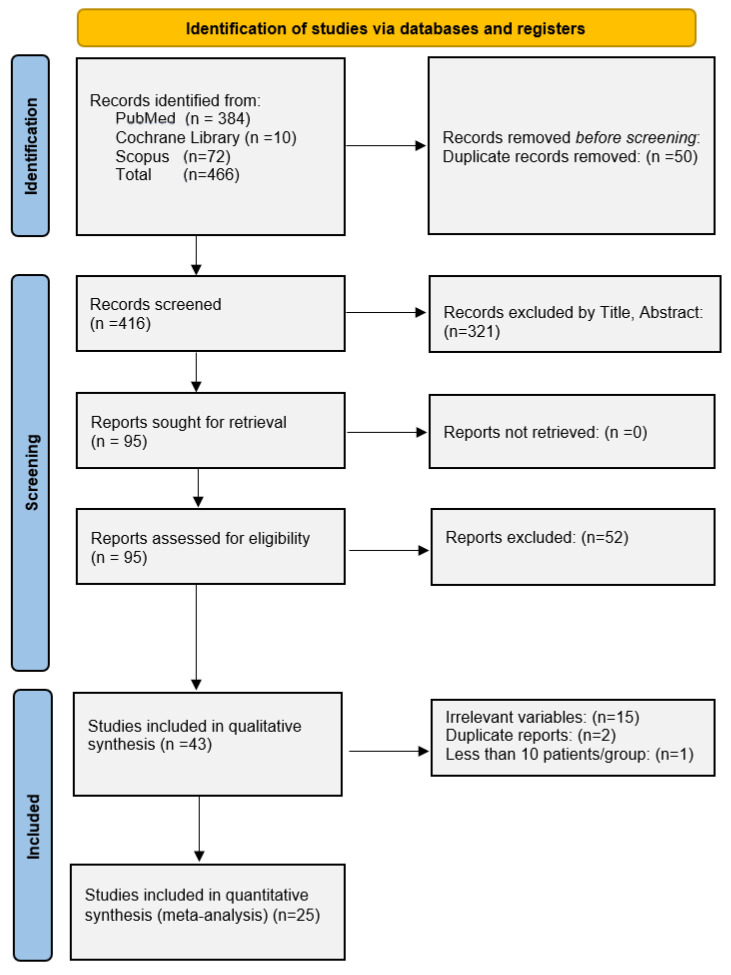
PRISMA Flow Chart.

The target population was adult patients diagnosed with cutaneous melanoma before (PreCovid) or during the COVID-19 pandemic (PostCovid). The studies selected met the following inclusion criteria: (1) controlled studies; (2) evaluation of cutaneous melanoma; (3) reported data on melanoma characteristics from diagnoses; (4) reported data from Europe; and (5) publication in a peer-reviewed journal. We excluded studies of therapeutic regimens for melanoma, studies from outside Europe, and review articles, duplicate reports, studies with fewer than 10 patients in each comparison group, editorials, and correspondences (Figure 1).

Furthermore, to properly assess the effect of each pandemic phase, we resorted to a sub-analysis of the outcomes of interest recorded before the 1st lockdown (Precovid/Prelock), during the 1st lockdown period (Year 2020, Lock), after the 1st lockdown (Year 2020, Pand), and after the implementation of the vaccines (Year 2021/22, Vac). Since several studies reported outcomes that overlapped with the aforementioned periods, two more study groups were created to properly synthesize the available data. These consisted of data reported during the 1st lockdown period, the reporting of which extended over several pandemic months (LockPand), and data derived during the 1st lockdown and which extended over the pandemic and the vaccination group (LockPandVac) (Table 1).

### 2.3. Study Selection

Two reviewers (K.S. and N.B.) independently screened the retrieved database files and the full texts of potentially eligible studies for relevance. Disagreement was resolved by consensus.

### 2.4. Data Collection and Risk of Bias Assessment

Data extraction was conducted independently by the two aforementioned authors using a standardized form. Discrepancies were resolved by consensus. The reviewers extracted data, including the general study characteristics, population characteristics, and outcomes of interest. The primary outcome was the Breslow thickness of melanoma at excision. Secondary outcomes included the presence of ulceration and the American Joint Committee for Cancer (AJCC) tumor stage [11].

The quality of studies was evaluated using the Cochrane ROBINS-I tool for assessing bias in non-randomized studies.

In order to include more data in the analysis, we resorted to data transformation, using medians, interquartile ranges, ranges, and patient numbers, and imputed standard deviations (SDs) for those reported variables for which data were lacking [12,13]. These techniques have been established to provide accurate results, even though bias may have been introduced through their use [13].

### 2.5. Data Synthesis and Analysis

Meta-analysis of the outcomes of interest was performed when data were available from at least two studies. Mean differences (MDs) along with 95% confidence intervals (CIs) were calculated for the continuous variable (Breslow thickness), while odds ratios (ORs) with 95% CIs were calculated for dichotomous outcomes (tumor staging, ulceration). We fitted an inverse variance statistical approach for the continuous variable, while a Mantel–Haenszel model was used for the dichotomous ones. Due to the presence of significant heterogeneity in the design and sampling of the studies included, a random effects model was utilized for all outcomes of interest. The significance level was set at *p* ≤ 0.05. Subgroup and sensitivity analyses were additionally conducted to explore potential sources of heterogeneity across the different pandemic phases. Heterogeneity was assessed via Cochran’s Q and Higgins’s I^2^ statistics. Forest plots were generated to present the effect sizes of each study accompanied by the 95% CIs. Funnel plots were constructed to properly assess publication bias. Egger’s statistical test was performed when the number of studies analyzed permitted the calculation, without limiting its statistical power. The meta-analysis was conducted using the ‘meta’ package in R, version 4.2.1 (R Foundation for Statistical Computing, Austria) [14,15].

## 3. Results

The study selection process is summarized in Figure 1. From a total of 466 records, 25 studies were incorporated in our data analysis models [16,17,18,19,20,21,22,23,24,25,26,27,28,29,30,31,32,33,34,35,36,37,38,39,40].

### 3.1. General Study Characteristics

The 25 studies included were conducted in Italy (6), Ireland (4), Spain (4), Germany (2), Greece (1), the UK (1), Romania (1), Austria (1), Belgium (1), France (1), the Netherlands (1), and Switzerland (1), with one study containing data from six European hospitals. All of the studies were observational and published between 2020 and 2022 (Table 2).

The risk of bias was considered moderate, based on the quality of the studies. Publication bias was assessed by visual inspection of the funnel plots (Figures S1–S3). Relative symmetry was consistently observed. Egger’s test was performed for the outcomes of mean Breslow thickness and ulceration between the PreCovid and PostCovid groups (*p* = 0.76 and *p* = 0.26, respectively), and for the mean Breslow thickness for the PreLock and LockPand groups (*p* = 0.44), since its use for the rest of the investigated outcomes would have been statistically underpowered.

### 3.2. Patient Characteristics and Baseline Clinical Profile

The meta-analysis included a total of 32,231 patients; 18,192 patients were included in the PreCovid group and 14,129 in the PostCovid group. The individuals’ baseline characteristics are presented in Table 1. A gender comparison of the PreCovid and PostCovid groups could be made for nine of the studies, indicating reduced incidence of melanoma in males (OR = 0.92 (95% CI: 0.88–0.98), *p* = 0.006) during the pandemic. Nine studies reported the ages of the patients; with a standardized mean difference (SMD) = −0.064, there was no significant difference between the Pre- and PostCovid groups (*p* = 0.86). Finally, in the analysis on the effect of the diagnosis during the different pandemic phases, 18,192 patients were included in the Prelock, 1456 in the Lock, 2627 in the LockPand, 3777 in the Pandemic, 2592 in the LockPandVac, and 3714 in the Vaccination groups.

### 3.3. Outcomes

**Figure 2:**
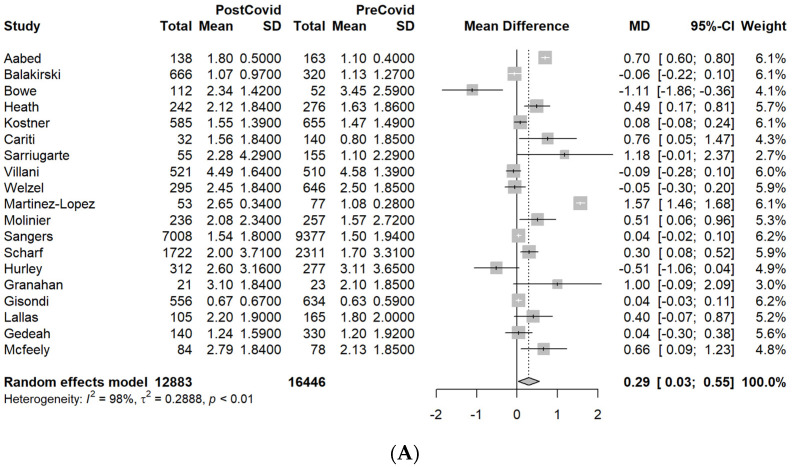

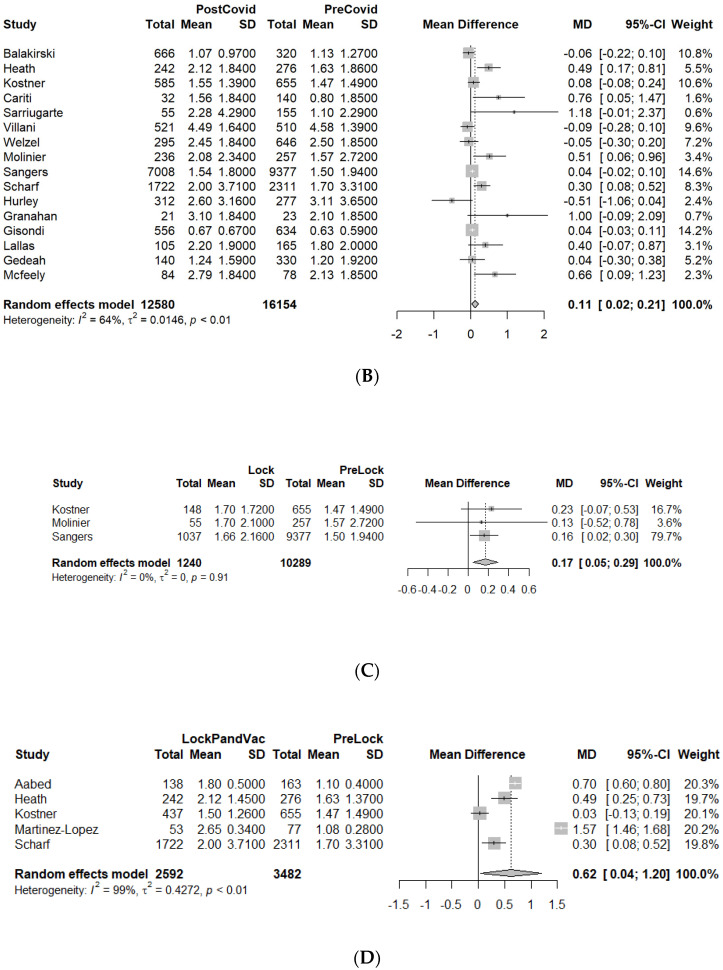
(**A**). Forest plot of Breslow thickness results for the PreCovid and PostCovid groups. (**B**). Forest plot of the Breslow thickness sensitivity analysis results for the PreCovid and PostCovid groups. (**C**). Forest plot of Breslow thickness results for the PreLock and Lock groups. (**D**). Forest plot of Breslow thickness results for the PreLock and LockPandVac groups.

**Figure 3:**
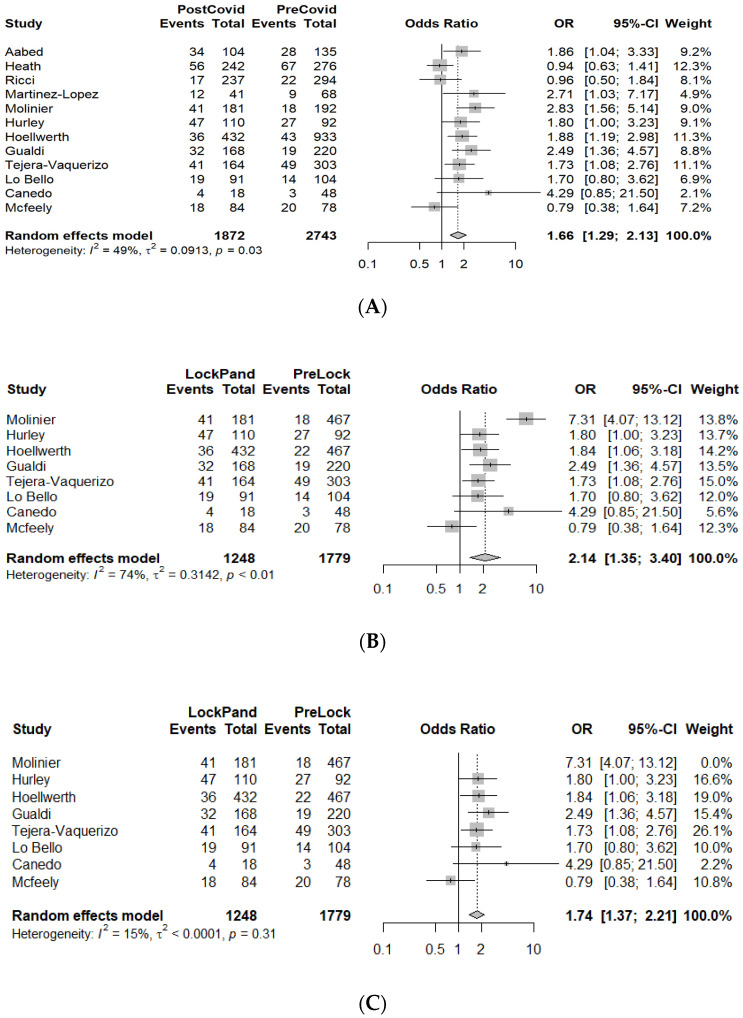
(**A**). Forest plot of ulceration rates for the PreCovid and PostCovid groups. (**B**). Forest plot of ulceration rates for the PreLock and LockPand groups. (**C**). Forest plot of ulceration rate sensitivity analysis results for the PreLock and LockPand groups.

**Figure 4:**
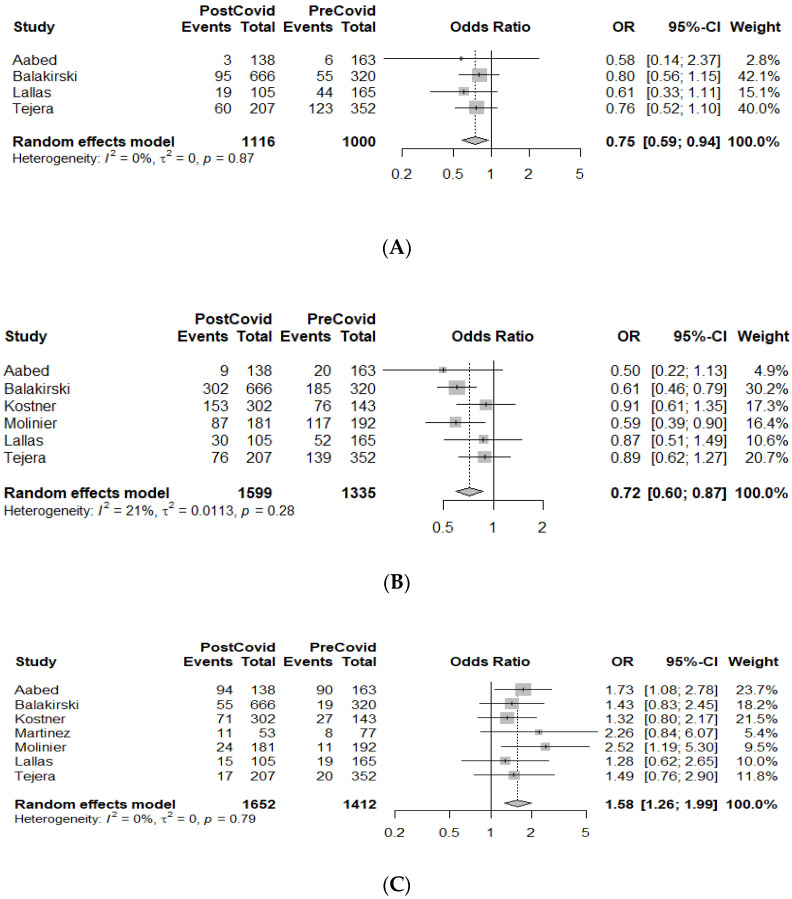
(**A**). Forest plot of AJCC Stage 0 results for the PreCovid and PostCovid groups. (**B**). Forest plot of AJCC Stage I results for the PreCovid and PostCovid groups. (**C**). Forest plot of AJCC Stage III results for the PreCovid and PostCovid groups.

The MDs and ORs (with 95% CIs) for the outcomes of interest (Breslow thickness, ulceration, and AJCC tumor stage) are presented as forest plots, along with core information from the meta-analysis (Figure 2, Figure 3 and Figure 4, Supplementary Materials Figures S1–S3).

#### 3.3.1. Breslow Thickness (mm)

A total of 19 studies reported the mean Breslow thicknesses of the diagnosed melanomas recorded during the PreCovid and PostCovid periods (*n* = 29,329 patients). We found a significant increase in Breslow thickness for the PostCovid group (MD = 0.29 mm (95% CI: 0.03–0.55 mm), *p* = 0.03, I^2^ = 97.7%), though there was considerable heterogeneity across the studies (Figure 2A). Thereafter, we performed a sensitivity analysis by removing the outliers and influential studies (with effect sizes so extreme that they differed significantly from the overall effect). The 16 studies included demonstrated the same trend towards thicker tumors in the PostCovid period (MD = 0.11 mm (95% CI: 0.02–0.21 mm), *p* = 0.017, I^2^ = 64.2%), with a substantial reduction in study heterogeneity compared with the main analysis (Figure 2B).

Focusing on the patients’ subgroups within the PostCovid period, we found a significant increase in Breslow thickness for the Lock compared to the PreLock group (MD = 0.17 (95% CI: 0.05–0.29), *p* = 0.006, I^2^ = 0%) (Figure 2C), based on evidence from three studies. Notably, the study of Sangers et al. exerted a sizeable influence in this analysis due to the large number of reported patients, though without being an outlier [35]. Moreover, a similar increase was also noticed when comparing five studies reporting on the LockPandVac compared with the PreLock group (MD = 0.62 (95% CI: 0.04–1.2), *p* = 0.035) (Figure 2D). Finally, three further analyses that compared the PreLock group with the LockPand (11 studies), Pand (2 studies), and Vac groups (5 studies) all failed to demonstrate significant Breslow thickness alterations.

#### 3.3.2. Ulceration

A total of 12 studies including *n* = 4615 patients reported comparisons of melanoma ulceration rates between the PreCovid and PostCovid periods. Our analysis showed a significant increase in the rate of ulcerated tumors in the PostCovid group (OR = 1.66 (95% CI: 1.29–2.13), *p* < 0.0001) (Figure 3A).

In addition, we analyzed the data for the subsections of the PostCovid period in order to determine which period had the most considerable impact in terms of the appearance of more neglected tumors presenting this malignant characteristic. A total of eight studies including 3027 patients reported on ulceration rates for the LockPand group, and the available evidence suggested a significant increase compared with the PreLock group (OR = 2.14 (95% CI: 1.35–3.40), *p* = 0.0012, I^2^ = 73.9%) (Figure 3B). A sensitivity analysis omitting the study of Molinier et al., which was an influential outlier, reached the same conclusion with dramatically reduced heterogeneity, improving confidence in the results (OR = 1.74 (95% CI: 1.37–2.21), *p* < 0.0001, I^2^ = 15.4%) (Figure 3C) [33]. Data extracted from three studies with 866 patients reporting on the LockPandVac group showed no differences compared to the PreLock group (OR = 1.52 (95% CI: 0.82–2.83), *p* = 0.19). The remaining data permitted no further analysis for the rest of the periods.

#### 3.3.3. AJCC Tumor Stage

A total of nine studies reported on the AJCC tumor staging of the melanomas diagnosed in the PreCovid and PostCovid periods (*n* = 3064 patients). Data from four studies that reported in situ melanomas revealed a significant reduction in the rate of Stage 0 tumor diagnoses in the PostCovid group (OR = 0.75 (95% CI: 0.59–0.94), *p* = 0.01) (Figure 4A). Similarly, data derived from six studies showed a reduction also in the rate of Stage I melanomas in the PostCovid group (OR = 0.72, (95% CI: 0.60–0.87), *p* = 0.0006) (Figure 4B). On the other hand, focusing on the more advanced melanomas, we found that the rate of diagnoses of Stage III melanomas was significantly higher in the PostCovid group (seven studies; OR = 1.58 (95% CI: 1.26–1.99), *p* < 0.0001) (Figure 4C). No statistically significant differences were observed for Stage II and Stage IV cancer patients after pooling effects from seven and six studies, respectively. Due to limited data availability, relevant subgroup analyses could not be performed.

## 4. Discussion

The purpose of the present meta-analysis was to summarize the available evidence on the impact of the COVID-19 pandemic on the management of patients with malignant melanoma in Europe by synthesizing data on Breslow thickness, ulceration, and tumor staging. Our findings support a significant trend towards clinically more advanced, thicker tumors with higher ulceration rates in the PostCovid group.

Meanwhile, several relevant observational studies from Europe with restricted numbers of patients and ambiguous outcomes pertaining to the impact of the pandemic on melanoma diagnosis and treatment have been published [41]. The findings of the present meta-analysis are indicative of the disruptive effect of the COVID-19 pandemic on European healthcare systems. The restrictions adopted across the continent had complex and diverse effects on morbidity from skin diseases. In particular, heavy restrictions on access to and the availability of specialized dermatology care services led to a reduction of more than 75% in dermatological activities [41]. As compared with most other medical specialties, this also included cancer consultations [42,43]. In addition, dermatologic patients were deterred from attending medical consultations amidst fears of viral transmission, with multiple reports commenting on the witnessed waves of skipped and postponed appointments [42]. Under the pressure of the pandemic, many patients discontinued treatments for chronic skin conditions, with a typical example being biologics for psoriasis [44]. However, the observed disruption in the provision of healthcare management in the case of cutaneous melanoma contradicts the updated guidelines of the relevant organizations, which proactively supported the strategy of undisrupted melanoma treatment, with deferrals considered only for early-stage melanomas [7,8,45].

A nationwide study on malignant diseases in Germany demonstrated that the number of patients with newly diagnosed cancer decreased during lockdown as compared with the pre-lockdown reference period; however, differentiating according to the anatomical site of tumor origin, skin cancers, including malignant melanoma, showed the greatest (−12.8%) and the only statistically significant decrease among all anatomical sites [42]. Similarly, in the subgroup analysis performed herein, the derived data from the lockdown period (for the Lock, LockPand, and LockPandVac groups) clearly indicated more advanced tumors in terms of histopathological depth and ulceration presence. Interestingly, this trend seemed to dissipate for the patients examined in the later periods (in the Pand and Vac groups), when the return to normality was almost established. The impact of the COVID-19 pandemic in preventive screening, as highlighted by the reduced numbers of patients in large campaigns, such as Euromelanoma, could account for this alteration [46]. This observation will be attested in the forthcoming years through assessment of the recorded alterations in melanoma-attributed mortality rates or the need for provision of systemic therapies for melanoma.

Aiming to properly portray the effect of the neglected melanomas on patient survival rates, Tejera-Vaquerizo et al. constructed an exponential growth model for melanoma to estimate tumor size after 1, 2, and 3 months of surgical delay, suggesting that delaying melanoma treatment by 1 month or longer increases the proportion of more advanced cases [47]. The proportion of patients with thick melanomas (>6 mm) increased from 6.9% in the initial study group to 21.9%, 30.2%, and 30.2% at 1, 2, and 3 months, respectively. Both 5- and 10-year disease-specific survival decreased by 14.4% in patients treated after a potential delay of 3 months.

This meta-analysis addresses the impact of the COVID-19 pandemic on cutaneous melanoma diagnosis. Among the strengths of this study is the rigorous methodology used: the analysis of a large sample size enabled reliable subgroup and sensitivity analyses to be performed as required. In addition, the different groups studied had similar baseline characteristics, thus limiting potential bias from known confounding factors with respect to the primary outcomes of interest. Finally, no significant publication bias was discovered, further enhancing the study outcomes.

The main limitation of the study is the notable degree of heterogeneity encountered in several of the comparisons. However, this was anticipated, as the data originated from different European countries with diverse healthcare systems and divergent populations regarding inherent melanoma risk factors. Moreover, not all outcomes of interest were uniformly reported in the included studies, which introduced an anticipated bias effect in the results of the present meta-analysis.

## 5. Conclusions

This meta-analysis has reported on the impact of COVID-19 restrictions on melanoma diagnosis in Europe, supporting a negative effect of the pandemic on prompt melanoma diagnosis. The evidence presented herein has implications for the future, as it shows the need for the continuation of screening procedures for the prompt diagnosis of melanoma, even in the case of emergency healthcare adaptations. Future studies will address the impact of advanced melanoma stage on patient characteristics, which is relevant to disease burden, as are the need for systemic therapy and survival rates.

## Figures and Tables

**Table 1:** Timelapse of the COVID-19 pandemic.

Era	PreCovid		PostCovid			
Period	Prelock		Lock	Pand	Vac	
**Year**	2019	2020			2021	2022
**Months**	January–December	January–February	March–May	June–December	January–December	January–to date

**Table 2:** Study characteristics.

	Author [Reference]	Year	Country	Period	Groups *	N	Age ^#^	Sex		Reported Outcomes
								M	F	
1	Aabed [16]	2022	Romania	January 2018–January 2020	PreLock	163	58.1 (16.3)	157	144	Breslow thickness Ulceration Tumor staging
				January 2020–January 2022	LockPandVac	138	58.8 (15.9)			
2	Balakirski [17]	2022	Germany	January–December 2019	PreLock	320	63.7 (17.7)	NR	NR	Breslow thickness Ulceration
				January–December 2020	LockPand	319	63.0 (19.4)			
				January–December 2021	Vac	347	65.7 (16.4)			
3	Bowe [18]	2022	Ireland	January–December 2019	PreLock	52	NR ^	73	90	Breslow thickness
				January–December 2020	LockPand	61				
				January–December 2021	Vac	51				
4	Granahan [23]	2022	Ireland	March–August 2019	PreLock	23	NR	NR	NR	Breslow thickness
				March–August 2020	LockPand	21				
5	Heath [25]	2022	UK	November 2018–March 2020	PreLock	276	NR	135	141	Breslow thickness Ulceration
				March 2020–March 2021	LockPandVac	242		118	124	
6	Hurley [27]	2022	Ireland	March–December 2019	PreLock	277	68.5 (25–96) ^##^	137	140	Breslow thickness Ulceration
				March–December 2020	LockPand	312	63.1 (24–91) ^##^	146	166	
7	Kostner [28]	2022	Switzerland	February 2019–March 2020	PreLock	655	64.0 (15.4)	741	497	Breslow thickness Tumor staging
				March–June 2020	LockPand	148				
				June 2020–April 2021	Pandemic + Vac	437				
8	Martinez-Lopez [31]	2022	Spain	March 2019–March 2020	PreLock	77	63.3 (1.9) ^###^	43	34	Breslow thickness Ulceration Tumor staging
				March 2020–March 2021	LockPandVac	53	65.0 (2.3) ^###^	23	30	
9	Molinier [33]	2022	France	March–October 2019	PreLock	257	NR	NR	NR	Breslow thickness Ulceration Tumor staging
				March–May 2020	Lock	55				
				May–October 2020	Pand	181				
10	Ricci [34]	2022	Italy	January–March 2020	PreLock	158	NR	NR	NR	Breslow thickness Ulceration
				March–May 2020	Lock	34				
				May–June 2020	Pand	45				
				January–June 2021	Vac	294				
11	Sangers [35]	2022	Netherlands	January 2019–March 2020	PreLock	9377	62.8 (15.0)	4704	4673	Breslow thickness
				March–May 2020	Lock	1037	61.5 (16.0)	495	542	
				June–October 2020	Pand	3532	63.1 (15)	1727	1805	
				April–July 2021	Vac	2439	63.5 (15)	1131	1308	
12	Sarriugarte [36]	2022	Spain	March–October (2018, 2019)	PreLock	155	NR	NR	NR	Breslow thickness
				March–October 2020	LockPand	55				
13	Scharf [37]	2022	6 European Centres	2019–2020	PreLock	2311	NR	NR	NR	Breslow thickness
				2020–2021	LockPandVac	1722				
14	Villani [39]	2022	Italy	2018	PreLock	216	55.4	13	17	Breslow thickness
				2019	PreLock	294	59.2	21	23	
				2020	LockPand	233	55.9	27	33	
				2021	Vac	288	57.3	22	25	
15	Weltzel [40]	2022	Germany	January 2019	PreLock	327	NR	NR	NR	Breslow thickness
				January 2020	PreLock	319				
				January 2021	Vac	295				
16	Fernández Canedo [20]	2021	Spain	April–August 2019	PreLock	48	NR	NR	NR	Ulceration
				April–August 2020	LockPand	18				
17	Cariti [19]	2021	Italy	May–June 2017	PreLock	51	61.0	31	20	Breslow thickness
				May–June 2018	PreLock	41	62.0	20	21	
				May–June 2019	PreLock	48	61.0	31	17	
				May–June 2020	LockPand	32	55.0	16	16	
18	Gedeah [21]	2021	Belgium	March–December 2018	PreLock	169	NR	NR	NR	Breslow thickness
				March–December 2019	PreLock	161				
				March–December 2020	LockPand	140				
19	Gisondi [22]	2021	Italy	March–October 2019	PreLock	634	61.0 (3.6) ^###^	351	283	Breslow thickness
				March–October 2020	LockPand	556	62.2 (3.6) ^###^	314	242	
20	Gualdi [24]	2021	Italy	March–July 2017–2019	PreLock	220	NR	262	271	Ulceration
				March–July 2020	LockPand	168				
21	Hoellwerth [26]	2021	Austria	March–June 2018	PreLock	428	61.0	228	200	Ulceration
				March–Jun 2019	PreLock	505	60.0	260	245	
				March–Jun 2020	LockPand	432	63.0	233	199	
22	Lallas [29]	2021	Greece	2016–2019	PreLock	165	58.7 (15.1)	140	130	Breslow thickness Tumor staging
				2020	LockPand	105	51.1 (11.4)			
23	Lo Bello [30]	2021	Italy	March–December 2019	PreLock	104	NR	NR	NR	Breslow thickness Ulceration
				March–December 2020	LockPand	91				
24	McFeely [32]	2021	Ireland	2019	PreLock	78	68.5 ^####^	73	89	Breslow thickness Ulceration
				2020	LockPand	84	75.5 ^####^			
25	Tejera-Vaquerizo [38]	2021	Spain	March–June 2019	PreLock	303	64.0 (16.4)	NR	NR	Ulceration
				March–June 2020	Lock	164	62.9 (16.7)			

* Period definitions: see Table 1. ^ NR: Not reported. ^#^ If not otherwise indicated, mean age (standard deviation) is reported. Otherwise: ^##^ Mean (range), ^###^ Mean (standard error of the mean), ^####^ Median.

## Supplementary Materials

The following supporting information can be downloaded at: https://www.mdpi.com/article/10.3390/cancers14246085/s1, Table S1: PRISMA checklist; Figure S1: (A) Funnel plot of Breslow thickness results for the PreCovid and PostCovid meta-analysis. (B) Funnel plot of Breslow thickness results for the PreCovid and PostCovid sensitivity analysis. (C) Funnel plot of Breslow thickness results for the PreLock and Lock analysis. (D) Funnel plot of Breslow thickness results for the PreLock and LockPandVac analysis; Figure S2: (A) Funnel plot of ulceration rates for the PreCovid and PostCovid meta-analysis. (B) Funnel plot of ulceration rates for the PreLock and LockPand meta-analysis. (C) Funnel plot of ulceration rates for the PreLock and LockPand sensitivity analysis; Figure S3: (A) Funnel plot of AJCC Stage 0 results for the PreCovid and PostCovid meta-analysis. (B) Funnel plot of AJCC Stage I results for the PreCovid and PostCovid meta-analysis. (C) Funnel plot of AJCC Stage III results for the PreCovid and PostCovid meta-analysis.
